# Spica Casting Results in More Unplanned Reoperations than Elastic Intramedullary Nailing: A National Analysis of Femur Fractures in the Preschool Population

**DOI:** 10.5435/JAAOSGlobal-D-20-00169

**Published:** 2020-10-01

**Authors:** Konstantin Brnjoš, David K. Lyons, Max J. Hyman, Neeraj M. Patel

**Affiliations:** From the Ann & Robert H. Lurie Children's Hospital of Chicago, Chicago, IL.

## Abstract

**Background:**

Spica casting and elastic stable intramedullary nailing (ESIN) are treatment options for femur fractures in preschool-age children. Clinical practice guidelines are only of moderate or limited strength, which may lead to variation in practice. The purpose of this study was to compare the revision surgery rate in young children undergoing these procedures.

**Methods:**

The Pediatric Health Information System, a database of 49 children hospitals, was queried for patients aged 3 to 6 years undergoing spica casting or ESIN for a diaphyseal femur fracture. ESIN removal was not considered an unplanned revision surgery because the indication for removal could not be determined in the database. Univariate analysis was followed by multivariate regression.

**Results:**

Analysis included 4,059 subjects with a mean age of 3.9 ± 1.1 years. Unplanned revision surgery was done in 227/2,878 children (8%) with a spica cast and 35/1,181 (3%) of those with ESIN (*P* < 0.01). Approximately 2% of children in each cohort underwent ESIN or open reduction and internal fixation as a revision procedure (*P* = 0.35). In multivariate analysis, spica casting resulted in 4.4 times higher odds of unplanned revision surgery than ESIN (95% confidence interval [CI], 2.9–6.7; *P* < 0.01). In the spica cast cohort, each year of increasing age resulted in 1.3 times higher odds of revision surgery (95% CI, 1.1–1.6; *P* < 0.01). Children who were aged 5 or 6 years at the time of spica casting had 1.9 times higher odds of requiring a subsequent procedure compared with 3- and 4-year-olds (95% CI, 1.3–2.7; *P* < 0.01).

**Conclusions:**

In this large, nationally representative cohort, spica casting resulted in higher odds of requiring an unplanned revision surgery than ESIN, when nail removal was not included as an unplanned procedure. Spica casting in 5- and 6-year-olds yielded higher odds of revision surgery. Regardless of whether spica casting or ESIN is chosen as the initial treatment, 2% of patients will subsequently undergo ESIN or open reduction and internal fixation as a revision procedure.

Femoral shaft fractures are a relatively common injury treated by pediatric orthopaedic surgeons. The optimal treatment of these fractures in preschool-age children is the subject of debate. Traditionally, these patients were treated with a hip spica cast. However, the development of improved instrumentation has made elastic stable intramedullary nailing (ESIN) a reliable alternative in some children. Some studies have suggested an increasing trend toward surgical management of femur fractures in the preschool population.^1,2^

A variety of factors guide treatment decisions. These include fracture pattern, age, weight, concurrent injuries, and parental caretaking considerations. The American Academy of Orthopaedic Surgeons (AAOS) Clinical Practice Guidelines (CPG) demonstrate moderate evidence supporting spica casting for children aged 6 months to 5 years with a diaphyseal femur fracture. They also note limited evidence to support flexible intramedullary nailing to treat children aged 5 to 11 years diagnosed with diaphyseal femur fractures.^3^ The preschool-age population lies at the intersection of these recommendations, which are of moderate strength at best.

Spica casting and ESIN each have inherent advantages and disadvantages. Some surgeons may consider the ease of postoperative care with ESIN when compared with spica casting in preschool-age children. Spica casting may also result in increased time away from work and school.^4,5^ ESIN may yield lower rates of malunion, earlier ambulation, and improved functional outcomes compared with spica casting^6,7^ but is a more invasive procedure, can require a second operation for implant removal, and likely has higher hospital-related costs.

Considering this equipoise and the paucity of strong evidence to guide decision-making, controversy exists regarding the optimal approach in preschool-age children. One factor that may help surgeons in treatment selection is an improved understanding of the revision surgery rate for each technique. Therefore, the purpose of this study was to compare frequency of unplanned revision surgery in preschool-age children undergoing spica casting and elastic IM nailing in a large, nationally representative population.

## Methods

The Pediatric Health Information System (PHIS), a national administrative database consisting of 49 children hospitals in the United States, was used for this study. The database contains inpatient, emergency department, ambulatory surgery, and observation unit data for hospitals affiliated with the Children's Hospital Association (CHA). Data quality is verified between the CHA and affiliated institutions and is subject to several reliability and validity checks before inclusion in the database. Because all information is deidentified, institutional review board approval was not required. Permission to report the data was obtained from the CHA.

The PHIS database was queried for patients between 3 and 6 years of age treated for a femur fracture between 2010 and 2017. This age range was chosen based, in part, on the previous literature on the subject so that the study would be comparable with previous reports.^6,8,9^ The database was first queried for all Current Procedural Terminology (CPT) and International Classification of Diseases (ICD) codes related to a femur fracture in this age group, yielding 5,521 subjects. Next, all patients with procedural or diagnosis codes indicative of nondiaphyseal fractures were excluded (ie, proximal, distal, or physeal fractures). Each subject's longitudinal data, including encounters unrelated to the femur fracture, were investigated, and those with diagnosis or procedural codes related to a pathologic fracture, neuromuscular disease, or syndrome were also excluded. Finally, all CPT and ICD procedural codes other than spica casting and ESIN were excluded. The final list of included codes was CPT codes (27500, 27502, 27506, 29305, and 29325) and ICD 9 and 10 procedural codes (7905, 7915, 0QH806Z, 0QH836Z, 0QH906Z, 0QH936Z, 0QS806Z, 0QS836Z, 0QS906Z, 0QS936Z, 0QH846Z, 0QH946Z, 0QS846Z, 0QS946Z, 0QS8XZZ, 0QS9XZZ, 2W36X2Z, 2W37X2Z, 2W3LX2Z, and 2W3MX2Z). This yielded a final cohort of 4,059 subjects with both a diagnosis and procedural code indicative of spica or ESIN for a femoral shaft fracture, each of whose data were again reviewed manually to confirm appropriate inclusion.

Demographic information was collected as was any administrative and clinical information related to the patient's index encounter. Each subject's longitudinal data were then analyzed individually for encounters related to the index femur fracture. Attention was paid to subsequent procedures related to the original femur fracture. Of note, although information regarding implant removal was collected for subjects that had undergone ESIN, this was not included as an unplanned procedure because elective removal cannot be distinguished from unplanned removal in the database.

Statistical analysis was completed with SPSS for Macintosh, version 24.0 (IMB, Armonk, NY). Standard descriptive statistics were calculated for demographic variables. Specifically, means are reported as ±SD. The Mann-Whitney *U* test was used for comparison of nonparametric variables. Categorical variables were analyzed using the chi-squared or Fisher exact tests, as appropriate. Means were compared with independent samples *t*-tests. Univariate analysis was followed by purposeful entry logistic regression to weigh clinical relevance while respecting statistical significance and adjusting for confounders. Odds ratios are reported with 95% confidence intervals (CIs). A significance threshold of *P* < 0.05 was applied for all statistical tests.

## Results

A total of 4,059 subjects met the criteria for inclusion. The mean age of this population was 3.9 ± 1.1 years, and 3,011 (74%) were male subjects. Spica casting was done in 2,878 cases (71%) and ESIN in 1,181 (29%). Patients who underwent spica casting were, on average, younger than those receiving ESIN (*P* < 0.001), and a slightly higher proportion were male patients (*P* < 0.001; Table 1). The median length of hospitalization was 1 day (interquartile range 1 day) for those who underwent spica casting compared with 2 days (interquartile range 1 day) for ESIN (*P* < 0.01).

**Table 1:** Comparison of Spica Casting and Elastic Stable Intramedullary Nailing (ESIN)^a^

Variable	Spica Cast	ESIN	*P* Value
n	2,878 (70.9)	1,181 (29.1)	NA
Age (yrs)	3.5 ± 0.8	4.9 ± 1.0	
Age 3 and 4	2,526 (87.8)	374 (31.7)	<0.001
Age 5 and 6	352 (12.2)	807 (68.3)	<0.001
Sex			
Male	2,191 (76.1)	820 (69.4)	<0.001
Female	655 (22.8)	351 (29.7)	
Unknown	32 (1.1)	10 (0.8)	
Race/ethnicity			
White	1,952 (67.8)	777 (65.8)	0.43
Black	467 (16.2)	215 (18.2)	
Asian	58 (2.0)	25 (2.1)	
Native American	18 (0.6)	7 (0.6)	
Pacific Islander	7 (0.2)	2 (0.2)	
Other	303 (10.5)	102 (8.6)	
Unknown	73 (2.5)	53 (4.5)	
Insurance			
Government	1,482 (51.5)	622 (52.7)	0.50
Private	1,185 (41.2)	484 (41.0)	
Other	211 (7.3)	75 (6.4)	
Median hospitalization (d) (interquartile range)	1 (1)	2 (1)	<0.001
Emergency department visit	52 (1.8)	13 (1.1)	0.10
Readmission	73 (2.5)	24 (2.0)	0.34
Observation unit stay	40 (1.4)	22 (1.9)	0.26
Refracture	20 (0.7)	5 (0.4)	0.32
Unplanned revision surgery	227 (7.9)	35 (3.0)	<0.001

NA = not applicable

a Values reported as n (%) or mean ± SD unless otherwise noted.

Unplanned procedures were subsequently done in 227/2,878 children (8%) who underwent spica casting compared with 35/1,181 (3%) of those treated with ESIN (*P* < 0.01). As previously mentioned, this did not include implant removal, which was done in 687/1,181 (58%) of patients who initially received ESIN. Most reoperations done in children with a spica cast were closed reductions (Table 2). The procedures done at the time of revision surgery were significantly different for patients who were initially treated with spica casting versus those who received ESIN (*P* < 0.001; Table 3). Although 8% of subjects who initially underwent spica casting ultimately required another operation, 68/2,878 (2%) ultimately had ESIN or open reduction with internal fixation (ORIF) as their revision operation. This frequency was similar in those who were initially treated with ESIN (22/1,181 = 2%; *P* = 0.35).

**Table 2:** Characteristics of Subjects Undergoing Unplanned Revision Surgery^a^

Variable	Unplanned Revision Surgery	No Revision Surgery	*P* Value
n (% of entire cohort)	262 (6.5)	3,797 (93.5)	NA
Age (yrs)	3.9 ± 1.0	3.9 ± 1.1	0.30
Sex			
Male	197 (75.2)	2,814 (74.1)	0.86
Female	63 (24.0)	943 (24.8)	
Unknown	2 (0.8)	40 (1.1)	
Race/ethnicity			
White	181 (69.1)	2,548 (67.1)	1.00
Black	44 (16.8)	638 (16.8)	
Asian	6 (2.3)	77 (2.0)	
Native American	1 (0.4)	24 (0.6)	
Pacific Islander	0 (0.0)	9 (0.2)	
Other	27 (10.3)	378 (10)	
Unknown	3 (1.1)	123 (3.2)	
Insurance			
Government	146 (55.7)	1,958 (51.6)	0.31
Private	96 (36.6)	1,573 (41.4)	
Other	20 (7.6)	266 (7.0)	
Index procedure			
Spica cast	227 (86.6)	2,651 (69.8)	<0.001
ESIN	35 (13.4)	1,146 (30.2)	
Median hospitalization (d) (interquartile range)	1 (1)	1 (1)	0.30

NA = not applicable

a Does not include patients that underwent implant removal after ESIN.

Values reported as n (%) or mean ± SD unless otherwise noted.

**Table 3:** Comparison of Unplanned Revision Surgery Procedures Done^a^

Variable	Spica Cast	ESIN
n	227	35
Closed reduction	149 (65.6)	8 (22.9)
ESIN	50 (22.0)	19 (54.3)
Open reduction, internal fixation	18 (7.9)	3 (8.6)
Epiphysiodesis/hemiepiphysiodesis	6 (2.6)	3 (8.6)
Limb lengthening	2 (0.9)	0 (0.0)
Unknown	2 (0.9)	2 (5.7)

ESIN = elastic stable intramedullary nailing

a *P* < 0.001.

Values reported as n (%).

Multivariate logistic regression was then done to adjust for any confounding variables. After controlling for multiple factors, spica casting resulted in 4.4 times higher odds of unplanned revision surgery than ESIN (95% CI, 2.9–6.7; *P* < 0.01). No additional variables retained statistical significance. The details of this multivariate model are shown in Table 4.

**Table 4:** Predictors of Unplanned Revision Surgery in Multivariate Regression

Variable	Odds Ratio	95% CI	*P* Value
Spica cast	4.4	2.9-6.7	<0.001
Sex	0.8	0.2-3.5	0.74
Geographic region	1.3	0.8-2.2	0.23

CI = confidence interval

Subgroup analysis was then done for patients who were treated with a spica cast. Within this study arm, 44/352 patients (13%) who were aged 5 or 6 years underwent an unplanned revision surgery compared with 183/2,526 subjects (7%) who were aged 3 or 4 years (*P* = 0.001). Multivariate analysis of the spica cast cohort revealed that each year of increasing age resulted in 1.3 times higher odds of revision surgery (95% CI, 1.1–1.6; *P* < 0.01). Furthermore, children who were aged 5 or 6 years at the time of spica casting had 1.9 times higher odds of requiring a subsequent procedure compared with 3- and 4-year-olds (95% CI, 1.3–2.7, *P* < 0.01). This multivariate model is displayed in Table 5.

**Table 5:** Predictors of Unplanned Revision Surgery After Initial Spica Casting

Variable	Odds Ratio	95% CI	*P* Value
Age (continuous)	1.3	1.1-1.6	<0.001
Age 5 or 6 yrs^a^	1.9	1.3-2.7	0.001
Sex	1.1	0.3-4.8	0.90
Geographic region	0.6	0.3-1.1	0.09

CI = confidence interval

a Age was first added to the model as a continuous variable, then separately as a categorical variable in another model.

## Discussion

Spica casting and ESIN are viable options for diaphyseal femur fractures in young children. However, equipoise persists regarding the benefits of one over the other in the preschool-age population. In this large, nationally representative analysis, we found that spica casting resulted in 4.4 times higher odds of requiring an unplanned procedure than ESIN when nail removal was not counted as an unplanned revision surgery. Most of the unplanned reoperations in the spica cohort were recasting procedures. Regardless of whether spica casting or ESIN was the original treatment, ESIN or ORIF was the revision procedure for 2% of each group. When further analyzing only children who underwent spica casting, increasing age resulted in higher odds of requiring a subsequent procedure.

Although both ESIN and spica casting are reasonable options in this age group, each has its advantages and disadvantages. Potential advantages of ESIN are quicker mobilization and return to activities, more stable fixation with possibly less malunion and greater ease of care.^6,8,10,11^ Disadvantages are that it is a more invasive surgical procedure that may require a second operation for implant removal and that the estimated immediate costs are higher compared with spica.^9,12,13^ Spica casting is less invasive and has a lower estimated immediate cost. However, it carries a risk of skin complications, extols a notable burden of care, and may have financial and social implications because of parents missing work.^4,14^

Previous retrospective studies comparing the outcomes of spica casting and ESIN in this age range did not find notable differences in the rates of revision surgery between the two procedures, although these studies included fewer centers and less subjects. Heffernan et al reported 4 total unplanned reoperations in a cohort of 215 patients, all of them in the spica group. The authors also noted longer times to independent ambulation and return to full activities in patients that underwent spica casting.^8^ Jauquier et al reported insignificant differences in complication rates between spica (2/19) and ESIN (4/27) groups, although it is unclear whether this study was adequately powered to detect such differences. In addition, patients were only aged 1 to 4 years.^10^ Of the 52 patients who underwent spica casting in the study by Assaghir, 13% underwent recasting under anesthesia, 17% had redressing for pressure sores, and 31% required wedging for unacceptable angulation. On the other hand, 4% of patients with ESIN had painful nail tips that required trimming and 2% (one patient) had nail exteriorization. ESIN resulted in better fracture length and alignment, as well as earlier weight-bearing and return to nursery.^6^ Finally, Ramo et al^11^ reported similar rates of unplanned revision surgery in their cohort of 262 patients, with 4% in both groups returning to the operating room. Although these studies are important additions to the literature on this topic, they may be underpowered to detect statistically notable differences in the revision surgery rate. By using a national database, we were able to analyze 4,059 subjects and identify differences between those undergoing spica casting and ESIN. In addition, concern has previously been voiced regarding the concern for limb length discrepancy because of overgrowth after treatment of femur fractures in this young population.^15^ Although the current study was unable to capture detailed clinical information because of database limitations, guided growth procedures or limb lengthening were only ultimately done in 8/2,878 patients (0.3%) who previously underwent spica casting and 3/1,181 (0.3%) who initially had ESIN. This suggests that the frequency of clinically relevant limb length discrepancy requiring surgical intervention may be relatively low.

The current AAOS CPG on femoral shaft fractures are of limited or moderate strength. The guidelines recommend spica casting in patients aged 6 months to 5 years and flexible intramedullary nailing for patients aged 5 to 11 years, with the level of evidence ranging from II to V for these recommendations.^3,16,17^ A multicenter review examining management of pediatric femoral shaft fractures before and after release of AAOS CPG found considerable variability in treatment and adherence to the guidelines. The authors also noted an increased trend toward surgical management in patients younger than five years despite the guidelines.^2^ Spica casting and ESIN may both be viable options in preschool-age children, which is the age group where these recommendations overlap. However, given that these guidelines are, at best, of moderate strength, additional research is necessary to determine the true benefits of one treatment over the other in this age group. The present study suggests that spica casting may result in a higher rate of unplanned revision surgery, especially in older children. Notably, this does not include nail removal. This must be explained to families because many patients with ESIN may require a second operation for implant removal, even if it is a planned procedure.

Notable limitations exist to this study, including its retrospective design. The PHIS database is administrative in nature and provides minimal clinical information regarding fracture characteristics, injury mechanism, or other pertinent clinical findings that could have impacted treatment choice or subsequent risk of revision surgery. Furthermore, although surgeons around the United States may have varying reasons for selecting a particular treatment or doing a secondary procedure, these indications cannot be deduced from the database. For example, it was impossible to determine whether nail removal was elective or unplanned. Although some variability surely exists, a large, nationally representative cohort likely provides sufficient power to lessen its impact and enhance generalizability. In addition, the database query was based on CPT and ICD coding, and although the PHIS takes steps to ensure data quality, there remains the possibility that coding errors could affect our results.

In conclusion, both spica and ESIN are reasonable treatment options for femoral shaft fractures in the preschool population. Although both modalities may ultimately lead to acceptable outcomes, each has its risks and benefits. In the present analysis of a large, nationally representative cohort, spica casting resulted in 4.4 times higher odds of undergoing an unplanned procedure, most commonly recasting. Regardless of whether spica casting or ESIN is chosen as the initial treatment, 2% of patients subsequently underwent ESIN or ORIF as a revision procedure. Older children who underwent spica casting were especially at risk for requiring a subsequent procedure, suggesting that ESIN may be worth consideration in these patients with the acknowledgement that many will undergo elective, planned nail removal. Although our results can be used to counsel families for shared decision-making, prospective comparison of spica casting and ESIN in preschool-age children would ultimately provide stronger evidence for treatment selection.

## References

[r-1] Levels of evidence are described in the table of contents. In this article, reference 5 is a level II study. References 1, 2, 6, 7, 8, 9, 10, 13, and 14 are level III studies. References 4, 11, and 12 are level IV studies. Reference 15 is a letter to an editor. References 3 and 16 are clinical practice guidelines. Reference 17 is a review article.

[1] Alluri RK Sabour A Heckmann N Hatch GF VandenBerg C: Increasing rate of surgical fixation in four- and five-year-old children with femoral shaft fractures. J Am Acad Orthop Surg 2019;27:e24-e32. 30180090 10.5435/JAAOS-D-17-00064

[2] Roaten JD Kelly DM Yellin JL: Pediatric femoral shaft fractures: A multicenter review of the AAOS clinical practice guidelines before and after 2009. J Pediatr Orthop 2019;39:394-399. 31393292 10.1097/BPO.0000000000000982

[3] Jevsevar DS Shea KG Murray JN Sevarino KS: AAOS clinical practice guideline on the treatment of pediatric diaphyseal femur fractures. J Am Acad Orthop Surg 2015;23:e101. 26507293 10.5435/JAAOS-D-15-00523

[4] Hughes BF Sponseller PD Thompson JD: Pediatric femur fractures: Effects of spica cast treatment on family and community. J Pediatr Orthop 1995;15:457-460. 7560034 10.1097/01241398-199507000-00009

[5] Flynn JM Luedtke LM Ganley TJ: Comparison of titanium elastic nails with traction and a spica cast to treat femoral fractures in children. J Bone Joint Surg Am 2004;86:770-777. 15069142 10.2106/00004623-200404000-00015

[6] Assaghir Y: The safety of titanium elastic nailing in preschool femur fractures: A retrospective comparative study with spica cast. J Pediatr Orthop B 2013;22:289-295. 23511585 10.1097/BPB.0b013e328360266e

[7] Saseendar S Menon J Patro D: Treatment of femoral fractures in children: Is titanium elastic nailing an improvement over hip spica casting? J Child Orthop 2010;4:245-251. 21629377 10.1007/s11832-010-0252-z PMC2866851

[8] Heffernan MJ Gordon JE Sabatini CS: Treatment of femur fractures in young children: A multicenter comparison of flexible intramedullary nails to spica casting in young children aged 2 to 6 years. J Pediatr Orthop 2014;35:126-129. 10.1097/BPO.0000000000000268 25105984

[9] Lewis RB Hariri O Elliott ME Jo CH Ramo BA: Financial analysis of closed femur fractures in 3- to 6-year-olds treated with immediate spica casting versus intramedullary fixation. J Pediatr Orthopaedics 2019;39:e114-e119. 10.1097/BPO.0000000000001253 30234705

[10] Jauquier N Doerfler M Haecker FM Hasler C Zambelli PY Lutz N: Immediate hip spica is as effective as, but more efficient than, flexible intramedullary nailing for femoral shaft fractures in pre-school children. J Child Orthop 2010;4:461-465. 21966311 10.1007/s11832-010-0279-1 PMC2946524

[11] Ramo BA Martus JE Tareen N Hooe BS Snoddy MC Jo CH: Intramedullary nailing compared with spica casts for isolated femoral fractures in four and five-year-old children. J Bone Joint Surg Am 2016;98:267-275. 26888674 10.2106/JBJS.O.00706

[12] Simanovsky N Tair MA Simanovsky N Porat S: Removal of flexible titanium nails in children. J Pediatr Orthop 2006;26:188-192. 16557132 10.1097/01.bpo.0000218534.51609.aa

[13] Levy JA Podeszwa DA Lebus G Ho CA Wimberly RL: Acute complications associated with removal of flexible intramedullary femoral rods placed for pediatric femoral shaft fractures. J Pediatr Orthop 2013;33:43-47. 23232378 10.1097/BPO.0b013e318279c544

[14] Difazio R Vessey J Zurakowski D Hresko MT Matheney T: Incidence of skin complications and associated charges in children treated with hip spica casts for femur fractures. J Pediatr Orthop 2011;31:17-22. 21150727 10.1097/BPO.0b013e3182032075

[15] Price CT: The conclusions reached by MJ Heffernan et al, may be incorrect (MJ Heffernan et al, “Treatment of femur fractures in young children: A multicenter comparison of flexible intramedullary nails to spica casting in young children aged 2 to 6 years”). J Pediatr Orthop 2016;36:e38. 26214330 10.1097/BPO.0000000000000613

[16] Kocher MS Sink EL Blasier RD: American Academy of Orthopaedic Surgeons clinical practice guideline on treatment of pediatric diaphyseal femur fracture. J Bone Joint Surg Am 2010;92:1790-1792. 20660244 10.2106/JBJS.J.00137

[17] Flynn JM Schwend RM: Management of pediatric femoral shaft fractures. J Am Acad Orthop Surg 2004;12:347-359. 15469229 10.5435/00124635-200409000-00009

